# CRITIC–Entropy-Weighted TOPSIS-Based Mix Ratio Optimization of Carbide Slag–Polypropylene Fiber-Modified Expansive Soil

**DOI:** 10.3390/ma19183967

**Published:** 2026-09-18

**Authors:** Junhua Chen, Xiulin Wei, Yuzi Nie, Aijun Chen, Xiong Shi

**Affiliations:** 1 School of Architecture and Transportation Engineering, Guilin University of Electronic Technology, Guilin 541004, China; jhchan@guet.edu.cn (J.C.); nieyuzhi0605@163.com (Y.N.); caj3026@guet.edu.cn (A.C.); shixiong@guet.edu.cn (X.S.); 2 School of Civil Engineering, Central South University, Changsha 410075, China

**Keywords:** swelling–shrinkage behavior, mechanical properties, CRITIC–entropy weight combination weighting method, TOPSIS

## Abstract

To address expansive soil swelling–shrinkage distress and the limitations of single carbide slag (brittle failure), pure fiber (limited strength), and cement (high carbon emissions), this study develops a multi-indicator optimization method for carbide slag–polypropylene fiber composite improvement. Twenty-five full-factorial tests were conducted under three schemes: single slag, single fiber, and composite improvement. Six indicators (USR, LSR, VSR, cohesion, internal friction angle, UCS) were used to construct a CRITIC–entropy–TOPSIS model. The new insight brought by this research lies in coupling combination weighting with TOPSIS to eliminate the deviation of one-sided optimal formulas biased toward swelling inhibition or strength growth obtained by conventional single weighting and conventional range analysis, filling the gap of a multi-objective evaluation system balancing indicator conflict and dispersion, and realizing multi-index balanced optimization. The optimal C8P0.3 (8% slag + 0.3% fiber) has a relative closeness of 0.93. Compared to raw soil, USR drops by 77.07%, and cohesion and UCS increase by 164.30% and 71.91%; compared to C8P0, cohesion and UCS increase by 8.56% and 10.16%, relieving brittleness. XRD/SEM reveal that slag hydration forms rigid C-S-H networks while fibers create flexible 3D networks, jointly achieving rigid–flexible synergy that fills pores and bridges cracks. This work provides an optimization approach for low-carbon expansive subgrade stabilization.

## 1. Introduction

Expansive soil is rich in hydrophilic minerals such as montmorillonite and illite. It swells and softens upon wetting and shrinks and hardens upon drying. This swelling–shrinkage behavior often causes landslides and collapses in subgrades, slopes, and foundations. The problem is particularly severe in regions with pronounced wetting–drying cycles. It results in economic losses of tens of billions of yuan annually [1]. To meet the long-term stability and durability requirements of subgrade engineering, expansive soil must be improved. The aim is to simultaneously enhance its swelling–shrinkage resistance and mechanical performance. These are the two core technical dimensions for evaluating the improvement effect.

Based on the fundamental principles of soil improvement, methods can be divided into two categories: physical improvement and chemical improvement [2]. Chemical improvement commonly employs cement [3] lime [4], fly ash [5], blast furnace slag [6], carbide slag [7], bagasse ash [8], and other materials. Among them, fly ash, slag, bagasse ash, and carbide slag are all industrial wastes. Studies have shown that these waste materials can reduce the clay fraction. They also improve plasticity indices. Moreover, they inhibit swelling through ion exchange and cementation. In physical improvement, traditional rigid materials [9,10] and anchor reinforcement techniques [11,12] are relatively mature. Fiber reinforcement technology [13,14,15], however, is attracting increasing attention. During swelling, fibers generate shear stress with the soil. This exerts a restraining effect. Considering that natural fibers are prone to degradation, using synthetic fibers to improve expansive soil can sustain the improvement effect over a longer period. Regardless of the improvement method employed, the evaluation of engineering performance involves multi-dimensional indicators such as swelling–shrinkage behavior and mechanical properties. This makes the determination of the improvement mix proportion essentially a multi-objective trade-off decision problem.

Regarding the mix proportion study of composite improved expansive soil, most scholars adopt the stepwise optimization method [16,17]. Some scholars further employ the response surface methodology or mixture design to explore factor interactions [18,19,20,21,22]. However, the above methods share common deficiencies. The stepwise optimization method struggles to quantify the synergistic effects of multi-factor interactions on multiple indicators, and the optimal mix proportion determined based on a single indicator or empirical judgment cannot guarantee comprehensive optimality across multiple indicators. While the response surface methodology can reveal interaction effects among factors, it fails to conduct systematic trade-offs and rankings among multiple indicators. On the other hand, current single evaluation methods for determining the optimal mix proportion of improved soil each have their own biases. The single CRITIC method, although capable of reflecting indicator conflict, may suppress the weights of swelling–shrinkage indicators that are correlated with strength; the single entropy weight method, while capturing data dispersion, neglects the inherent correlations among indicators and tends to amplify the contribution of a single strength indicator. The traditional orthogonal range analysis method determines the primary and secondary factors solely based on the range magnitude, and in practice it often prioritizes strength characteristics at the expense of swelling–shrinkage control. Therefore, it is imperative to introduce a multi-objective decision-making model and establish a multi-objective evaluation system that simultaneously accounts for indicator conflict and dispersion, while also enabling a quantitative comparison of the advantages of composite improvement over single improvement, thereby enhancing the scientific validity and reliability of mix proportion optimization at the methodological level.

To address the above problems, this study constructed a multi-indicator comprehensive evaluation model. This model couples the CRITIC–entropy weight combination weighting method with the conventional TOPSIS method. The TOPSIS method ranks evaluation objects based on relative closeness. It measures the distances to the best and worst solutions [23]. The TOPSIS method combined with CRITIC–entropy weight combination weighting overcomes the limitation of the traditional TOPSIS method. The traditional method cannot reflect variable correlation and importance. The CRITIC method measures the contrast intensity and conflict of indicators. It uses the standard deviation and correlation coefficient, respectively [24]. It reflects the competition between indicators. However, it is insensitive to the degree of dispersion within the indicator distribution. The entropy weight method quantifies the degree of variation of an indicator across alternatives based on information entropy [25]. However, it ignores the correlation structure between indicators.

The optimization of the expansive soil improvement mix proportion is essentially a multi-objective trade-off problem. It requires the coordination of swelling–shrinkage behavior and mechanical properties. It also needs to ensure sufficient discriminatory power of indicators for different alternatives. If mechanical indicators exhibit significant dispersion and swelling–shrinkage indicators are strongly correlated with them, single weighting methods can easily cause evaluation imbalance. The CRITIC method may excessively reduce the weights of correlated indicators. The entropy weight method may overly amplify the contribution of highly dispersed indicators. Complementarily integrating the two methods can account for both the indicator correlation structure and the discriminatory power of alternatives. This constructs a more robust comprehensive evaluation system. It effectively reflects the intrinsic connections between performance indicators. It also captures the performance differences among mix proportions. This method holds significant practical value. It enhances the durability of expressway subgrade in expansive soil regions. It reduces maintenance costs. It also promotes the high-value utilization of industrial solid waste. Accordingly, this study aims to develop a multi-indicator optimization method for carbide slag–polypropylene fiber composite improved expansive soil, achieving synergistic optimization of swelling–shrinkage inhibition and mechanical enhancement. The novelty lies in coupling CRITIC–entropy weight combination weighting with TOPSIS to eliminate the bias of single weighting and range analysis, and in quantitatively revealing the rigid–flexible synergy between slag hydration and fiber reinforcement. This work fills the gap of lacking a multi-objective evaluation system that simultaneously accounts for indicator conflict and dispersion for expansive soil mix proportion optimization. It provides a new technical pathway for the prevention and control of subgrade distress in problematic soils.

## 2. Materials and Methods

### 2.1. Raw Materials

The expansive soil used in the test was taken from the third bid section of the Enhu Road (Yongwu Road–Jinlun Road) project in Nanning. Its basic physical property indicators were determined through laboratory tests. The results are shown in Table 1. Carbide slag was purchased from Henan Wuhu Environmental Protection Technology Co., Ltd., Zhengzhou, China. Its main chemical composition is shown in Table 2. Polypropylene fiber was purchased from Shijiazhuang Chuangsheng Building Materials Technology Co., Ltd., Shijiazhuang, China. Its basic physical property indicators are shown in Table 3.

To better evaluate the reactivity of the carbide slag with expansive soil, its mineralogical and physicochemical characteristics were reviewed based on published studies, given that the slag used in this study is a typical by-product of the calcium carbide–acetylene production process. According to the literature [26,27,28], carbide slag is mainly composed of calcium hydroxide, with a content generally exceeding 80%. The calcium hydroxide phase supplies Ca^2+^ for cation exchange with montmorillonite interlayers and creates the alkaline environment needed for the pozzolanic reaction [29]. A minor amount of calcium carbonate is inert under test conditions. Fresh carbide slag contains little free calcium oxide, because hydrolysis directly yields calcium hydroxide rather than calcium oxide. The slag has a very fine particle size, with a D50 of 10–50 μm [29], which ensures high specific surface and rapid reaction kinetics with soil particles.

### 2.2. Experimental Design

According to the provisions on free swelling ratio in the Technical Code for Buildings in Expansive Soil Regions (DB45/T 396-2022) [30], combined with test results, the improved soil is classified as non-expansive when the carbide slag content reaches or exceeds 4%. Therefore, 4%, 6%, 8%, and 10% were selected as the carbide slag dosage range in this study. All physical and mechanical properties were determined according to the following Chinese standards: the unloaded swelling ratio (USR), linear shrinkage ratio (LSR), volumetric shrinkage ratio (VSR), cohesion, and internal friction angle (φ) were measured in accordance with JTG 3430-2020 (Test Methods of Soils for Highway Engineering) [31]; the unconfined compressive strength (UCS) was determined according to JTG 3441-2024 (Test Methods of Materials Stabilized with Inorganic Binders for Highway Engineering) [32]. Specifically, the direct shear tests, from which cohesion and φ were obtained, were performed using the consolidated quick shear method at a shear rate of 0.8 mm/min under vertical pressures of 100, 200, 300, and 400 kPa. The procedures specified in JTG 3430-2020 were strictly followed. All specimens were cured under standard conditions for 7 days prior to testing, in accordance with JTG/T F20-2015 [33].

To determine the optimum moisture content (OMC) and maximum dry density (MDD) for each carbide slag content, standard heavy compaction tests were conducted per JTG 3441-2024 [32] on mixtures with 0%, 4%, 6%, 8%, and 10% slag. Results show that as slag content increased from 0% to 10%, MDD decreased from 1.84 to 1.73 g/cm^3^, while OMC increased from 13.14% to 15.07%, attributed to the lower particle density and higher water absorption of Ca(OH)_2_ compared with soil particles. For fiber-reinforced specimens, given the very low fiber content and the inert, non-hydrating nature of polypropylene fibers, their influence on compaction characteristics is negligible. Therefore, for each level of slag content, the OMC and MDD from slag-only tests were used as the preparation reference for all specimens with the same slag content, regardless of fiber dosage.

To systematically compare the effectiveness of single improvement and composite improvement, three improvement schemes were designed in this study: (1) single carbide slag improvement, (2) single polypropylene fiber improvement, and (3) carbide slag–polypropylene fiber composite improvement. Full factorial design was used with a total of 25 pieces together; among them were the series C4P0~C10P0 and C0P0.1~C0P0.4 series as a single respectively improved control were used to quantify the superposition of composite modified efficiency. The research program of all mix proportions is presented in Table 4. For unloaded swelling and shrinkage tests, two replicate specimens were prepared per group; for UCS tests, three replicate specimens were prepared per group; and for direct shear tests, two replicate groups were set, each containing four specimens.

**Table 4:** Research program of all mix proportions.

Mix Proportion	Carbide Slag Content/%	Polypropylene Fiber Content/%	Mix Proportion	Carbide Slag Content/%	Polypropylene Fiber Content/%
C0P0	0	0	C6P0.3	6	0.3
C0P0.1	0	0.1	C6P0.4	6	0.4
C0P0.2	0	0.2	C8P0	8	0
C0P0.3	0	0.3	C8P0.1	8	0.1
C0P0.4	0	0.4	C8P0.2	8	0.2
C4P0	4	0	C8P0.3	8	0.3
C4P0.1	4	0.1	C8P0.4	8	0.4
C4P0.2	4	0.2	C10P0	10	0
C4P0.3	4	0.3	C10P0.1	10	0.1
C4P0.4	4	0.4	C10P0.2	10	0.2
C6P0	6	0	C10P0.3	10	0.3
C6P0.1	6	0.1	C10P0.4	10	0.4
C6P0.2	6	0.2	/	/	/

Note: “C” denotes carbide slag, and the number following it indicates the carbide slag content, and “P” denotes polypropylene fiber, and the number after it indicates the fiber content. All contents are expressed as the mass percentage of the stabilizer to dry soil. For example, “C4P0.4” represents a mix combination with 4% slag content and 0.4% fiber content. This table corresponds exactly to the mix proportion column in Table 5.

**Table 5:** Test data of evaluation indicators for composite improved soil (mean ± SD).

MP	USR /%	LSR /%	VSR /%	Cohesion /kPa	φ /°	UCS /MPa	MP	USR /%	LSR /%	VSR /%	Cohesion /kPa	φ /°	UCS /MPa
C0P0	32.71 +0.08	4.88 +0.06	6.48 +0.07	142.18 +2.43	24.17 +0.06	1.65 +0.04	C6P0.3	7.95 +0.06	1.86 +0.02	0.97 +0.02	386.25 +5.83	42.97 +0.06	2.80 +0.06
C0P0.1	31.28 +0.07	4.55 +0.04	5.90 +0.08	152.87 +5.50	26.54 +0.10	1.71 +0.07	C6P0.4	8.46 +0.06	2.14 +0.01	1.12 +0.03	378.44 +4.78	42.81 +0.03	2.69 +0.02
C0P0.2	28.20 +0.11	4.19 +0.06	5.67 +0.05	161.42 +5.78	28.35 +0.03	1.76 +0.02	C8P0	8.21 +0.06	2.27 +0.03	1.81 +0.03	346.15 +5.56	40.07 +0.03	2.56 +0.07
C0P0.3	26.91 +0.05	4.07 +0.04	5.51 +0.05	177.31 +5.29	29.63 +0.06	1.79 +0.01	C8P0.1	8.03 +0.06	2.19 0.03	1.62 +0.02	351.26 +5.52	41.38 +0.05	2.66 +0.06
C0P0.4	26.54 +0.04	3.78 +0.05	4.82 +0.03	181.82 +4.38	29.47 +0.08	1.94 +0.03	C8P0.2	7.77 +0.07	2.03 +0.02	1.27 +0.01	364.32 +5.37	42.12 +0.06	2.78 +0.05
C4P0	11.77 +0.02	3.57 +0.05	3.48 +0.02	242.46 +5.61	33.62 +0.02	2.21 +0.02	C8P0.3	7.50 +0.08	1.90 +0.01	1.19 +0.02	375.78 +5.37	43.25 +0.03	2.82 +0.04
C4P0.1	11.47 +0.07	3.51 +0.07	2.87 +0.02	250.19 +5.20	35.81 +0.08	2.43 +0.05	C8P0.4	7.81 +0.07	2.26 +0.02	1.35 +0.01	379.57 +6.42	42.65 +0.03	2.90 +0.05
C4P0.2	10.54 +0.03	3.32 +0.05	2.53 +0.03	263.74 +3.82	37.54 +0.02	2.67 +0.04	C10P0	4.23 +0.03	2.23 +0.04	1.4 +0.03	337.12 +4.14	39.39 +0.04	2.44 +0.07
C4P0.3	10.36 +0.06	3.18 +0.07	2.21 +0.03	287.33 +3.07	39.81 +0.02	2.71 +0.05	C10P0.1	4.13 +0.04	2.18 +0.04	1.27 +0.02	344.81 +2.97	40.98 +0.04	2.55 +0.05
C4P0.4	11.07 +0.09	3.30 +0.05	2.33 +0.04	291.65 +5.53	39.13 +0.01	2.79 +0.04	C10P0.2	3.71 +0.04	1.92 +0.03	1.11 +0.03	349.35 +5.66	41.83 +0.04	2.73 +0.05
C6P0	9.22 +0.07	2.23 +0.03	2.06 +0.03	328.14 +1.37	37.34 +0.01	2.53 +0.03	C10P0.3	3.54 +0.06	1.74 +0.03	0.84 +0.01	353.88 +2.96	42.17 +0.05	2.77 +0.04
C6P0.1	8.99 +0.08	2.19 +0.03	1.73 +0.04	351.83 +5.57	39.49 +0.01	2.67 +0.04	C10P0.4	3.80 +0.04	1.87 +0.02	0.95 +0.02	350.23 +5.52	41.54 +0.06	2.65 +0.04
C6P0.2	8.31 +0.07	1.99 +0.04	1.32 +0.04	369.97 +4.24	41.76 +0.01	2.71 +0.03	/	/	/	/	/	/	/

Note: Values are mean ± SD. The CV values for all indicators are below 10%, indicating good repeatability. In the table, “MP” denotes mix proportion; “USR” denotes unloaded swelling ratio; “LSR” denotes linear shrinkage ratio; “VSR” denotes volumetric shrinkage ratio; “*φ*” denotes internal friction angle; “UCS” denotes unconfined compressive strength.

All test results are reported as mean ± standard deviation (SD) based on replicate specimens. The coefficient of variation (CV) was calculated as the ratio of SD to the mean. One-way ANOVA followed by Duncan’s post hoc test was performed using SPSS 26.0 to evaluate the statistical significance of differences among mix proportions, with *p* < 0.05 considered statistically significant. The variance homogeneity test showed that all indicators satisfied homogeneity (*p* > 0.05).

### 2.3. Comprehensive Evaluation System Construction

The optimization of expansive soil improvement mix proportion is essentially a multi-objective trade-off problem. It must balance swelling–shrinkage inhibition and mechanical enhancement. Each indicator is also required to possess sufficient discriminatory power for different mix proportions. Therefore, this study established a multi-indicator decision-making model. This model couples the CRITIC–entropy weight combination weighting method with the conventional TOPSIS method. It sequentially includes three steps: standardization, combination weighting, and relative closeness ranking.

#### 2.3.1. Raw Data Processing

Six indicators were selected to construct the evaluation matrix: USR, LSR, VSR, cohesion, internal friction angle (IFA), and UCS. To eliminate dimensional effects, the linear proportion method was used for standardization [34].

For positive indicators (cohesion, IFA, UCS),

(1) $$x_{ij}^{\prime }=\frac{x_{ij}-minx_{j}}{maxx_{j}-minx_{j}}$$

For negative indicators (USR, LSR, VSR),

(2) $$x_{ij}^{\prime }=\frac{maxx_{j}-x_{ij}}{maxx_{j}-minx_{j}}$$

in which $x_{ij}^{\prime }\;$ is the standardized value of the *j*-th indicator for the *i*-th mix proportion; $x_{ij}$ is the original value; $\max x_{j}\;$ and $\min x_{j}\;$ are the maximum and minimum values of the *j*-th indicator, respectively. The standardized data range from 0 to 1. If a zero value exists, a non-negative shift of 0.0001 is applied [35].

#### 2.3.2. CRITIC Weighting Method

The CRITIC method measures indicator variability by the standard deviation. It measures conflict by the Pearson correlation coefficient. The product of the two yields the information content. After normalization, the weights are obtained.

(1) Variability calculation (represented by standard deviation):

(3) $$\sigma_{j}=\sqrt{\frac{l}{m-l}\sum_{i=1}^{m}{\left(x_{ij}^{\prime }-{\bar{x}}_{j}^{\prime }\right)}^{2}}$$

in which *m* is the number of samples, and $\;{\bar{x}}_{j}^{\prime \;}$ is the mean value of the *j*-th indicator. A larger $\;\sigma_{j}\;$ value indicates a greater potential weight.

(2) Conflict quantification:

The Pearson correlation coefficient $r_{jk}$ is used to construct the correlation matrix *R* among indicators:

(4) $$r_{jk}=\frac{\sum_{i=1}^{m}\left(x_{ij}^{\prime }-{\bar{x}}_{j}^{\prime }\right)\left(x_{ik}^{\prime }-{\bar{x}}_{k}^{\prime }\right)}{\sqrt{\sum_{i=1}^{m}{\left(x_{ij}^{\prime }-{\bar{x}}_{j}^{\prime }\right)}^{2}\sum_{i=1}^{m}{\left(x_{ik}^{\prime }-{\bar{x}}_{k}^{\prime }\right)}^{2}}}$$

(3) Information content calculation:

(5) $$\omega_{j}=\sigma_{j}\times \sum_{k=1}^{m}\left(1-r_{jk}\right)$$

(4) Weight normalization:

(6) $$\omega_{j}^{\prime }=\frac{\omega_{j}}{\sum_{j=1}^{n}\omega_{j}}$$

#### 2.3.3. Entropy Weight Method

The entropy weight method is an objective weighting method based on information entropy theory. A greater degree of indicator dispersion leads to a higher weight [36].

(1) Information entropy calculation:

(7) $$E_{j}=-k\sum_{i=1}^{n}p_{ij}{\ln p}_{ij}$$

in which $\;k=\frac{1}{\ln n}$, the proportion of the *i*-th ratio and the *j*-th indicator $\;p_{ij}=\frac{x_{ij}^{\prime }}{\sum_{i=1}^{n}x_{ij}^{\prime }}$, and n is the number of samples.

(2) Information utility:

(8) $$G_{j}=1-E_{j}$$

(3) Entropy weight:

(9) $$w_{j}=\frac{G_{j}}{\sum_{j=1}^{m}G_{j}}$$

#### 2.3.4. TOPSIS Method Based on CRITIC–Entropy Weight Combination Weighting

Based on the above analysis, using the CRITIC method or the entropy weight method alone may lead to evaluation bias. The former emphasizes inter-indicator conflict integration but suffers from information loss, while the latter relies solely on the degree of dispersion of the indicators themselves for weighting. It cannot reflect the structural correlation between swelling–shrinkage and mechanical indicators. To avoid the bias of single weighting methods and subjective preset weighting coefficients, the principle of minimizing the sum of squared deviations was adopted to determine the combination weights [36]. The CRITIC weight and the entropy weight were linearly combined.

The combination weight of each evaluation indicator is calculated using Equation (10):

(10) $$\lambda_{j}=\beta \omega_{j}^{\prime }+\left(1-\beta \right)w_{j}$$

in which *β* is the proportion of the CRITIC weight in the combination weight. The objective function of the sum of squared deviations *S*(*β*) is constructed:

(11) $$S\left(\beta \right)=\sum_{j=1}^{m}\left[{\left(\lambda_{j}-\omega_{j}^{\prime }\right)}^{2}+{\left(\lambda_{j}-\omega_{j}\right)}^{2}\right]$$

Substituting the linear weighting Formula (10) into (11), and through mathematical derivation, to minimize *S(β)*, the derivative with respect to *β* is taken and set to zero. The analytical solution for the optimal weight coefficient *β* is obtained as follows:

(12) $$\beta =\sum_{j=1}^{m}{\left(\omega_{j}^{\prime }-w_{j}\right)}^{2}/2\sum_{j=1}^{m}{\left(\omega_{j}^{\prime }-w_{j}\right)}^{2}=0.5$$

The above derivation shows the following. When the CRITIC method and the entropy weight method each account for 50% in the combination weight, the total deviation between the combination weight and the two single weights is minimized. This result objectively meets the dual information requirements of expansive soil improvement evaluation. The conflict relationship between indicators and the discriminatory power of the indicators themselves are equally important in this decision-making. It also avoids evaluation bias caused by subjective preset weight allocation.

Based on the combination weights, a weighted standardized matrix was constructed. The TOPSIS method was then applied to rank the alternatives. The positive ideal solution A^+^ and the negative ideal solution A^−^ are determined by Equations (13) and (14):

(13) $$A^{+}=\max\left(x_{ij}\right)$$

(14) $$A^{-}=\min\left(x_{ij}\right)$$

The Euclidean distances of each mix proportion from the positive and negative ideal solutions, Di^+^ and Di^−^, are, respectively,

(15) $$D_{i}^{+}=\sqrt{\sum_{j=1}^{n}{\left(x_{ij}-A_{j}^{+}\right)}^{2}}$$

(16) $$D_{i}^{-}=\sqrt{\sum_{j=1}^{n}{\left(x_{ij}-A_{j}^{-}\right)}^{2}}$$

The relative closeness is defined as

(17) $$C_{i}=\frac{D_{i}^{-}}{D_{i}^{+}+D_{i}^{-}}$$

## 3. Results

### 3.1. Test Results

Table 5 presents the specific data of the six evaluation indicators for each mix proportion, obtained from USR, shrinkage, direct shear, and UCS tests.

As shown in Table 5, one-way ANOVA followed by Duncan’s post hoc test confirmed that the differences among C8P0.3, C10P0.3, and C6P0.3 are statistically significant for UCS (*p* < 0.05) and cohesion (*p* < 0.05), while the differences in swelling–shrinkage indicators follow the same ranking trend. These results confirm that C8P0.3 achieves a statistically significant balanced optimum among the leading formulations.

### 3.2. Calculation Results of the Evaluation System

#### 3.2.1. CRITIC Weight Calculation Results

The Pearson correlation matrix of the six evaluation indicators is shown in Table 6. The results show strong positive correlations among USR, LSR, and VSR, with *r* = 0.863–0.986 and *p* < 0.01. Cohesion is also strongly correlated with UCS, with *r* = 0.923 and *p* < 0.01. The internal friction angle is strongly correlated with VSR and UCS.

**Figure 1:**
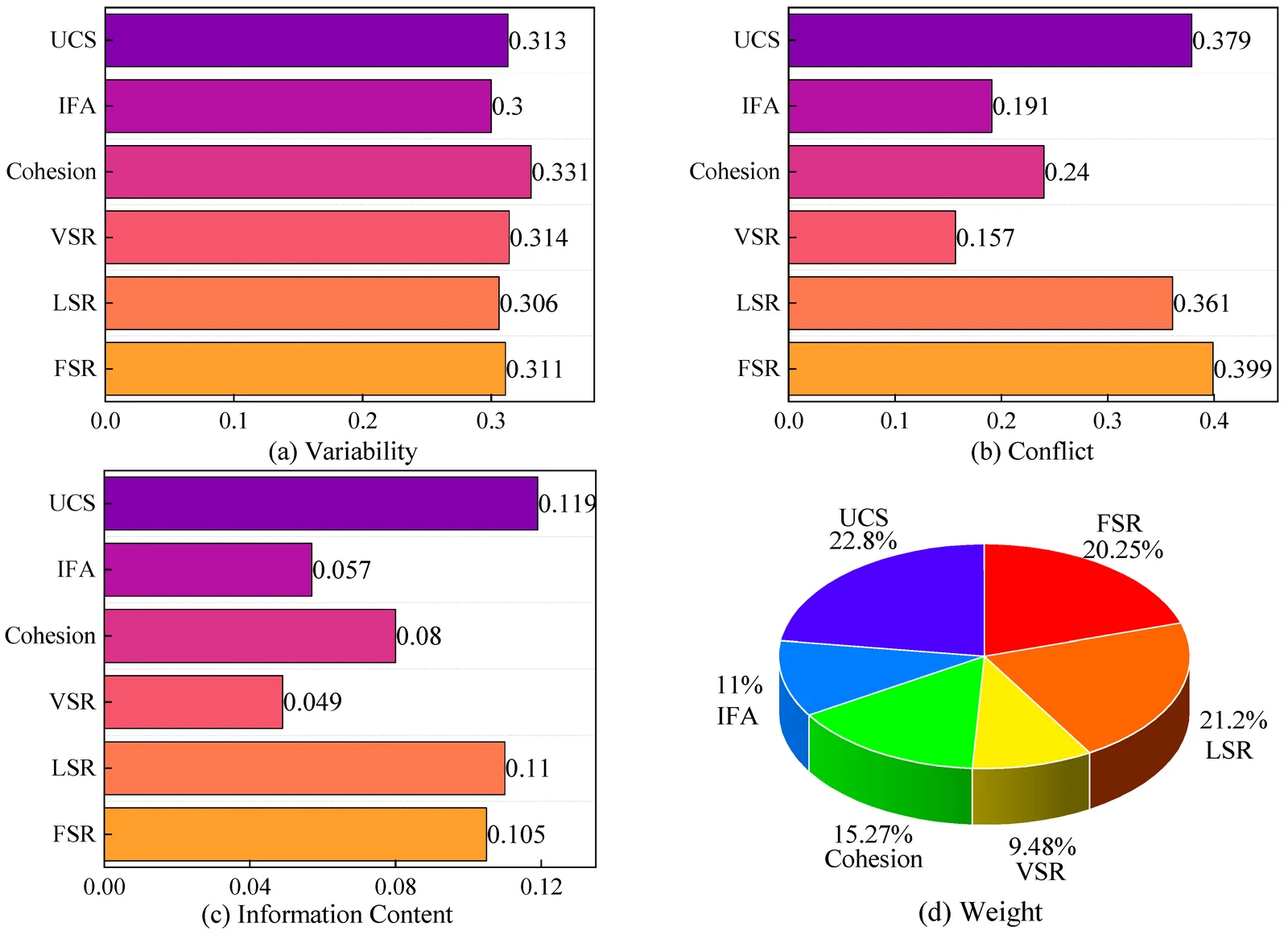
CRITIC weights of the evaluation indicators.

The CRITIC weight calculation results for the six evaluation indicators are shown in Figure 1.

The CRITIC method assigns weights by simultaneously quantifying indicator variability and conflict. This results in a weight structure dominated by strength and shrinkage indicators. The CRITIC calculation results, shown in Figure 1, indicate that UCS obtains the highest weight of 0.228 due to its highest conflict. It forms the core evaluation position together with LSR and USR. The sum of their three weights exceeds 0.6, dominating the direction of mix proportion optimization. Although cohesion ranks first in variability, its low conflict significantly limits its information load. Its weight falls into the secondary category. VSR and IFA have the lowest conflict or the smallest variability, respectively. Their weights are only 0.095 and 0.110, exerting minimal influence on the evaluation results. Evidently, the CRITIC method inherently favors the key strength and swelling–shrinkage items that reflect independent fluctuation characteristics of indicators. It fails to fully account for indicators with strong variability but weak conflict. Its weight allocation tends to weaken the balanced expression of multi-dimensional information to a certain extent. A risk of information loss exists when it is applied alone.

#### 3.2.2. Entropy Weight Calculation Results

**Figure 2:**
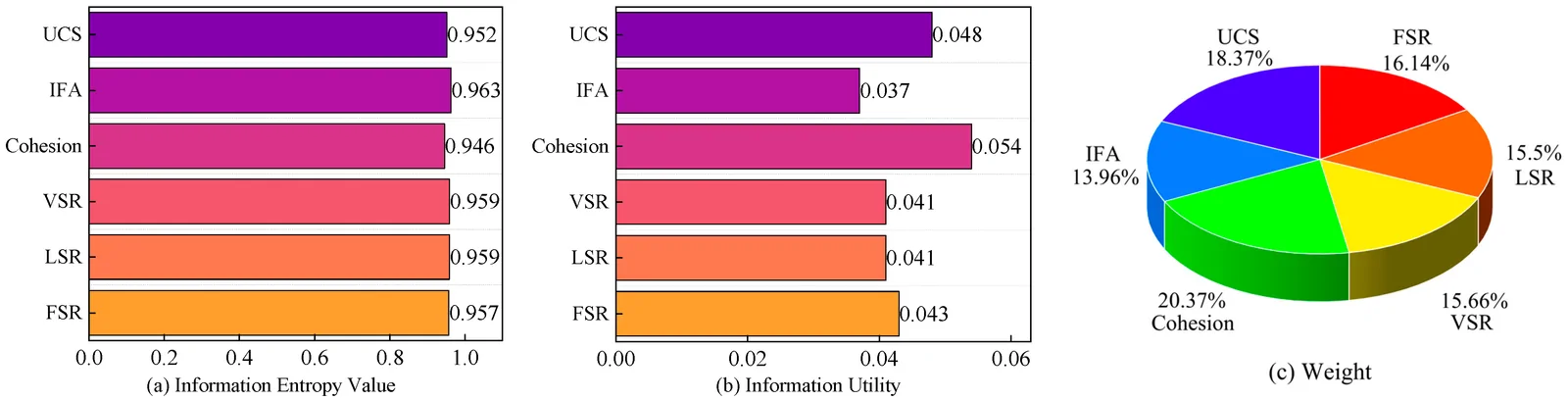
Entropy weight calculation results of the evaluation indicators.

The calculated entropy weights for the six evaluation indicators are shown in Figure 2.

The entropy weight method measures the degree of dispersion of indicator data through information entropy. A larger information utility indicates more significant data differences for that indicator. The assigned weight is then higher. As shown in Figure 2, cohesion has the lowest information entropy of 0.946 and an information utility of 0.054. It obtains the highest entropy weight of 0.204. This indicates its strongest variability and the most prominent ability to differentiate alternatives. UCS has an information entropy of 0.952, an information utility of 0.048, and an entropy weight of 0.184, ranking second in weight.

Among the three swelling–shrinkage indicators, the USR has an information entropy of 0.957, which is lower than 0.959 for both LSR and VSR. Its information utility is 0.043, slightly higher than 0.041 for the latter two. Therefore, its entropy weight of 0.161 is slightly higher than 0.150 for LSR and 0.157 for VSR. However, all three weights fall within a narrow range of 0.150–0.161, with balanced contributions. IFA has the highest information entropy of 0.963, an information utility of only 0.037, and the lowest entropy weight of 0.140, indicating the smallest data dispersion.

It can be seen that strength indicators, especially cohesion, are the key drivers of mix proportion optimization. IFA has weaker importance. When combining weights, the CRITIC method needs to be introduced to provide indicator conflict information, thereby optimizing the weight structure.

#### 3.2.3. Comprehensive Evaluation Results

**Figure 3:**
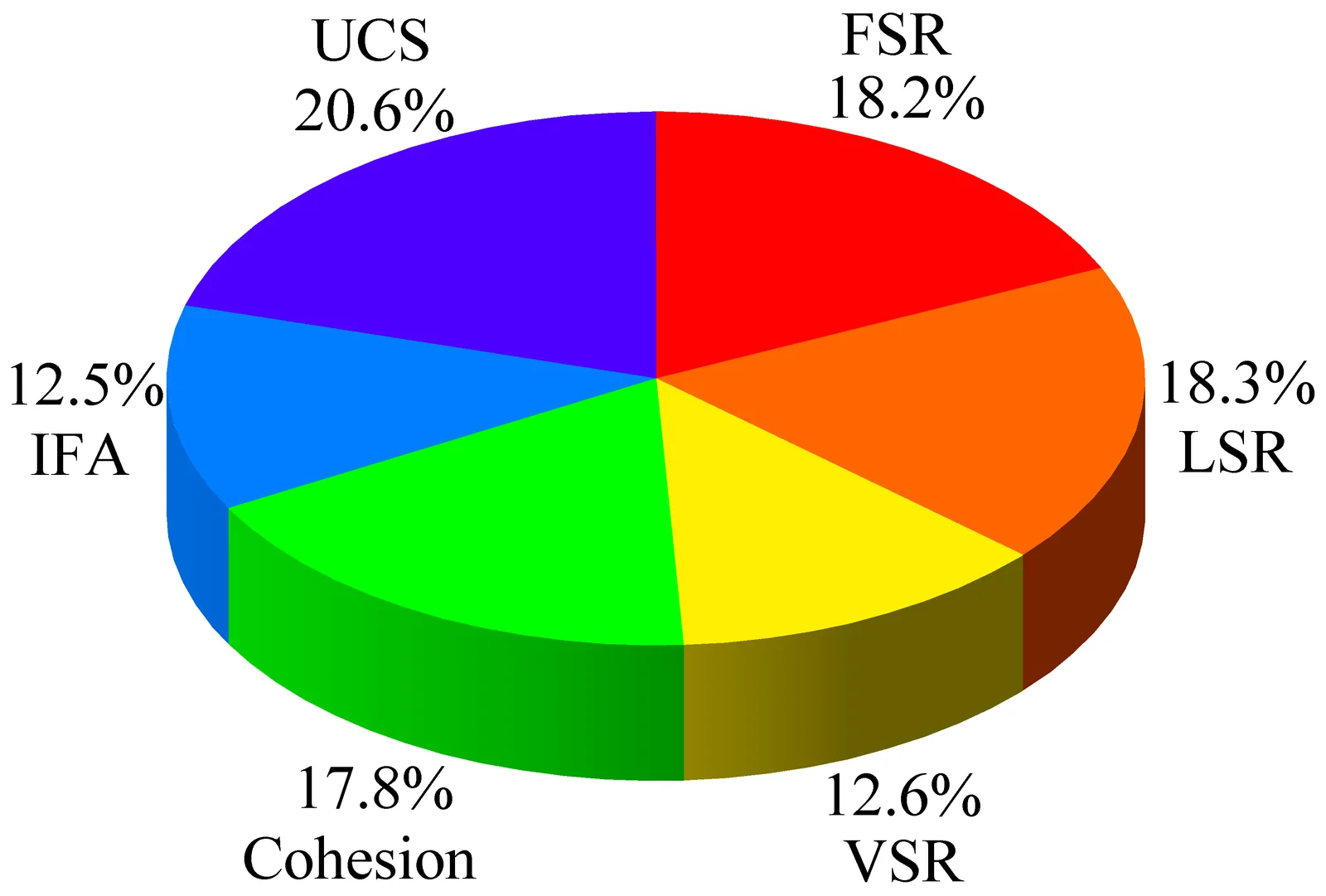
Combination weights of the evaluation indicators.

The calculated combination weights are shown in Figure 3.

All mix proportions are sorted in descending order of the $C_{i}$ value. The TOPSIS evaluation results are summarized in Table 7.

The TOPSIS evaluation results based on CRITIC–entropy weight combination weighting are shown in Table 7. Among all mix proportions, C8P0.3 achieves the highest relative closeness of 0.93, ranking first. This indicates the best comprehensive performance. C10P0.3 and C6P0.3 both have a relative closeness of 0.92, ranking second and third, respectively. These three together constitute the leading group. When the fiber content is fixed, as the carbide slag content increases from 0 to 8%, the relative closeness shows an overall upward trend. It peaks at C8P0.3. When the slag content reaches 10%, the relative closeness of C10P0.3 drops slightly to 0.92. Meanwhile, C10P0.4 drops to 0.88, ranking eighth. This indicates that excessive slag and fiber are both detrimental to synergistic performance improvement. The combination weighting TOPSIS method effectively identifies that mix proportions within the ranges of C6–C10 and P0.2–P0.4 generally have high relative closeness. Among them, the combination of 0.3% fiber and 8% carbide slag achieves a balanced optimization of strength and deformation. This provides a reference basis for mix proportion design.

To examine whether the small difference in relative closeness is sufficiently robust, a sensitivity analysis was performed by varying the CRITIC weight proportion from 40% to 60%. This range covers the entire spectrum from entropy-weight-dominated to CRITIC-weight-dominated weighting schemes. The TOPSIS relative closeness values and rankings were recalculated for each weight combination. The results show that C8P0.3 consistently ranks first across the entire range, while C10P0.3 and C6P0.3 always rank second and third, respectively. Their relative closeness values remain lower than that of C8P0.3 under all tested weight combinations. These results demonstrate that the optimal ranking is insensitive to the weighting scheme within a practically reasonable range, confirming that C8P0.3 is a robust optimum.

To further verify the synergistic effect, a two-way ANOVA was performed with carbide slag content (C) and fiber content (P) as fixed factors, including the C × P interaction term. The results show that for all six indicators, the main effects of C and P and the C × P interaction are statistically significant (*p* < 0.001). For example, for UCS, C: *F* = 1129.686, P: *F* = 126.203, and C × P: *F* = 8.597; for cohesion, C: *F* = 9439.999, P: *F* = 332.216, and C × P: *F* = 14.606; for USR, C: *F* = 371,359.306, P: *F* = 2794.781, and C × P: *F* = 878.509. This confirms that the composite improvement is a true synergistic interaction rather than a simple additive superposition.

### 3.3. Comparison of Optimal Mix Proportions from Different Evaluation Methods

The optimal mix proportions selected by each evaluation method and their corresponding six indicator values are presented in Table 8.

As shown in Table 8, C10P0.3 selected by the single CRITIC–TOPSIS method achieves the lowest unloaded swelling ratio of 3.54%, with a UCS of 2.77 MPa. C6P0.3 selected by the single entropy weight–TOPSIS method obtains the highest cohesion of 386.25 kPa, with a USR of 7.95%. C8P0.4 recommended by the conventional range analysis method achieves the highest UCS of 2.90 MPa, with a linear shrinkage ratio of 2.26%. C8P0.3 selected by the combination weighting method achieves the highest internal friction angle of 43.25°, and the fiber content is reduced from 0.4% to 0.3%.

### 3.4. Macro and Micro Analysis of the Optimal Mix Proportion

**Figure 4:**
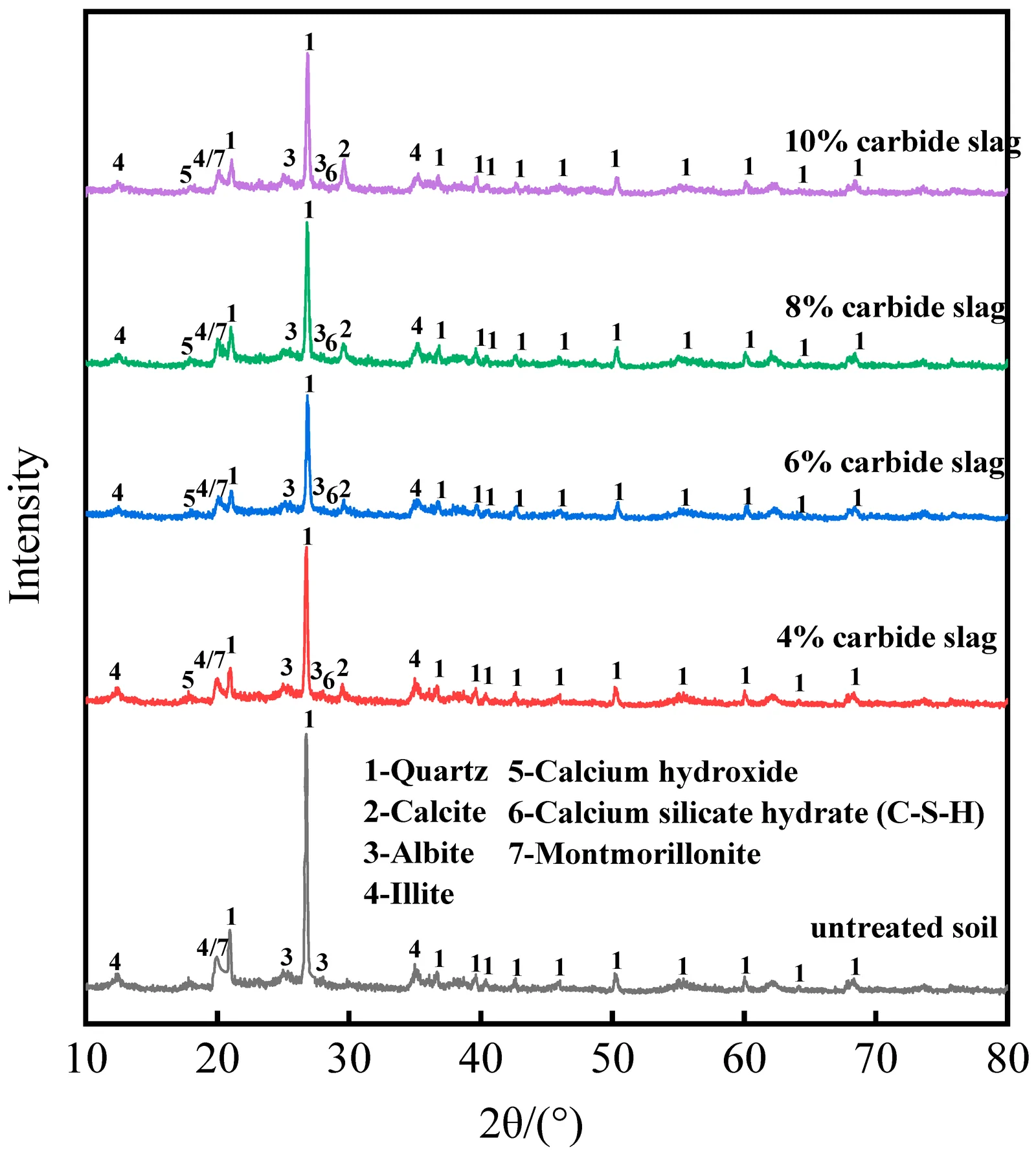
XRD patterns of soils improved with different carbide slag contents.

To elucidate the microstructural mechanisms underlying the macroscopic performance, XRD and SEM analyses were conducted on selected representative samples. XRD analysis was performed on five samples to reveal the hydration products and mineralogical evolution with increasing slag content: untreated expansive soil (C0P0) and carbide slag stabilized soils with 4%, 6%, 8%, and 10% slag content (C4P0, C6P0, C8P0, C10P0), respectively. Since polypropylene fibers do not participate in chemical reactions, XRD analysis was focused on the untreated and slag-treated soils. The XRD patterns of these samples are shown in Figure 4. The qualitative XRD analysis reveals that the untreated soil contains characteristic peaks of montmorillonite, illite, kaolinite, and quartz. According to published studies [37,38,39], Nanning Basin expansive soils typically contain approximately 9–25% montmorillonite, 13–29% illite, 32–57% kaolinite, and 10–15% quartz; the high montmorillonite content accounts for the raw soil’s high swelling potential. As the content of calcium carbide slag increases, the broad diffraction peak at approximately 29–30° (2θ) gradually intensifies, while the characteristic peak of montmorillonite weakens. This broad peak is mainly attributed to the formation of C-S-H gel, but it should be noted that calcite also has a characteristic peak at approximately 29.4° (2θ). The observed broad peak here may contain the superimposed contributions of C-S-H and calcite. The peak intensity monotonically increases with the content of calcium carbide slag, and at the same time, montmorillonite is consumed, while peaks of Ca(OH)_2_ and calcite appear, which is consistent with the gradual hydration and volcanic ash reaction. The observed calcite mainly originated from the original CaCO_3_ in the calcium carbide slag, possibly containing a small amount of Ca(OH)_2_ and carbonized contributions from atmospheric CO_2_. At the same time, both the soil and the calcium carbide slag contained Al_2_O_3_. Under alkaline conditions, it might form C-A-S-H or calcium aluminate hydrate, but XRD failed to clearly identify this.

**Figure 5:**
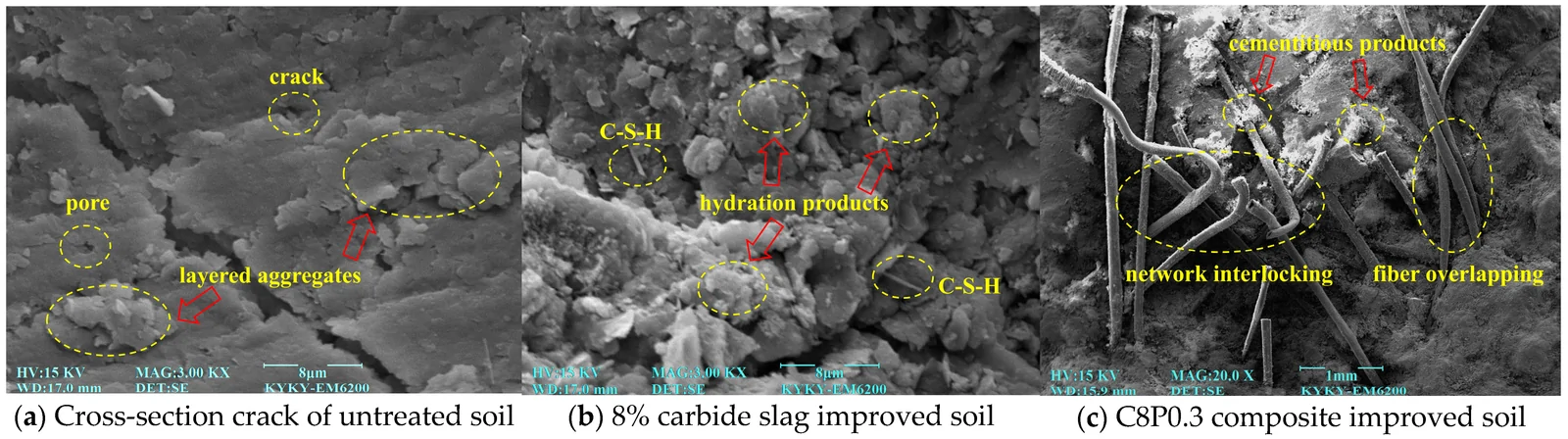
SEM images of untreated soil and improved soil.

Figure 5 shows the SEM images of untreated soil and improved soil obtained from three representative specimens (C0P0, C8P0, and C8P0.3). For each specimen, at least three randomly selected areas were examined to ensure representativeness. In Figure 5a, the untreated soil surface exhibits loose layered aggregates with well-developed pores and micro-cracks, and inter-particle bonding is weak. As shown in Figure 5b, after the incorporation of 8% carbide slag, the surface roughness of the soil increases, pores and cracks are markedly reduced, and hydration products and cementitious substances are visible on the particle surfaces. In Figure 5c, with the addition of 0.3% polypropylene fibers, the fibers are randomly distributed in the soil and intertwined with cementitious products into a network structure.

Macroscopically, the unloaded swelling ratio of C8P0.3 decreases from 32.71% for untreated soil to 7.50%, and the linear shrinkage ratio decreases to 1.90%. The cohesion and unconfined compressive strength of C8P0.3 increase by 164.30% and 71.91%, respectively, compared with untreated soil. Compared with the single carbide slag stabilized soil C8P0, C8P0.3 exhibits increases in cohesion and unconfined compressive strength of 8.56% and 10.16%, respectively.

## 4. Discussion

This study demonstrates that the TOPSIS model coupled with CRITIC–entropy weight combination weighting can effectively reconcile the conflict between swelling–shrinkage indicators and mechanical indicators. The recommended optimal mix proportion, C8P0.3 (8% carbide slag + 0.3% polypropylene fiber), does not pursue a single optimal indicator but rather achieves the overall optimum through multi-objective trade-off.

First, from the perspective of evaluation method applicability, single weighting methods possess inherent biases when optimizing expansive soil mix proportions. If the CRITIC method is used alone, its weight allocation relies on indicator conflict. Since unconfined compressive strength (UCS) has the strongest conflict with other indicators, this method tends to favor deformation control, resulting in the selection of C10P0.3. Although this mix achieves the lowest swelling–shrinkage ratios, its UCS is 1.77% lower than that of C8P0.3. When the entropy weight method is used alone, it is sensitive to data dispersion, and cohesion receives the highest weight, leading to the selection of C6P0.3. However, its unloaded swelling ratio (USR) deteriorates by 6.00% compared with C8P0.3. The conventional range analysis method determines primary and secondary factors solely based on range magnitude, often sacrificing swelling–shrinkage performance for strength. In contrast, the combination weighting method adopted in this study simultaneously accounts for indicator conflict and dispersion, enabling the optimal mix proportion C8P0.3 to achieve the best balance between strength and deformation.

**Figure 6:**
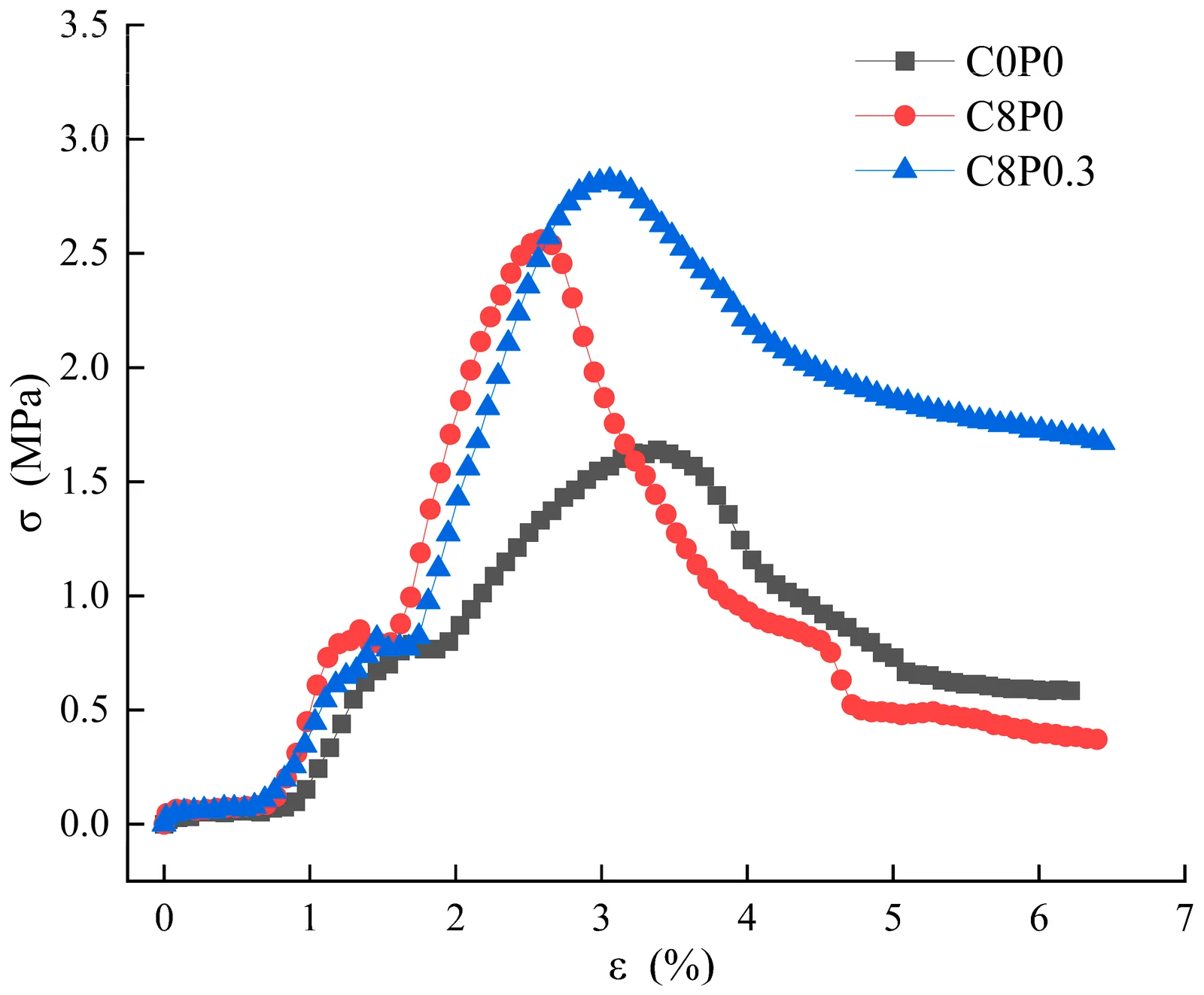
Stress–strain curve.

Second, the linkage between microstructural and macroscopic performance further reveals the fundamental reason why C8P0.3 exhibits excellent comprehensive performance. XRD and SEM analyses indicate the following. When carbide slag is added, the Ca^2+^ released from hydration replaces interlayer Na^+^ and K^+^ in the montmorillonite structure, reducing the diffuse double layer thickness and thereby lowering the swelling potential. The subsequent pozzolanic reaction between Ca(OH)_2_ and the dissolved silica/alumina generates C-S-H and C-A-H gels that fill pores and cement particles, providing additional resistance against swelling deformation. This explains why the USR of C8P0.3 drops from 32.71% (raw soil) to 7.50%, despite the raw soil’s significant montmorillonite content. This physically explains why the USR sharply decreases from 32.71% for untreated soil to 7.50%. However, in the single carbide slag stabilized soil (C8P0), the C-S-H network, although providing strength, forms a continuous rigid continuum that cannot prevent the propagation of microcracks. This is the microscopic origin of its pronounced brittle failure. When 0.3% polypropylene fibers are incorporated, the fibers are randomly distributed in the soil and intertwine into a three-dimensional flexible network, which forms a “rigid–flexible” interpenetrating structure with the C-S-H gel. Under initial loading, the rigid skeleton provides compressive bearing capacity; under later loading or during swelling–shrinkage deformation, the fibers bridge microcracks and provide interfacial friction, thereby resisting failure. This dual-network synergistic mechanism is macroscopically manifested as follows: compared with the single carbide slag stabilized soil (C8P0), the composite improved soil (C8P0.3) exhibits further increases in cohesion and UCS by 8.56% and 10.16%. From the stress–strain curve in Figure 6, it can be observed that the failure mode has changed from brittle to ductile. The residual strength of C8P0.3 at a strain of 6.2% is more than four times that of C8P0, and the decline after the peak is more gradual. These results confirm that the fiber network effectively bridges micro-cracks and maintains the post-peak load-bearing capacity, thereby transforming the failure mode from brittle to ductile.

Compared with traditional lime- or cement-stabilized expansive soils, the carbide slag used in this study is an industrial solid waste with significantly lower carbon emissions than cement production, meeting the requirements of low-carbon subgrade construction. Meanwhile, the incorporation of polypropylene fibers effectively compensates for the insufficient toughness of single carbide slag-stabilized soil. Although C8P0.3 performs excellently under the current test conditions, this study still has several limitations. Although carbide slag is an industrial solid waste, its low-carbon character should currently be regarded as a potential advantage rather than a proven conclusion; this study did not perform a full environmental assessment and did not consider the leaching behavior of carbide slag, transportation, waste preparation, or polypropylene fiber production. Previous studies have emphasized the importance of such assessments for solid-waste-based stabilization [40,41]; therefore, TCLP/SPLP leaching tests and life cycle assessment (LCA) are needed to verify its environmental safety and low-carbon benefits. Meanwhile, all experimental results are based on laboratory specimens cured under standard conditions for 7 d. In actual subgrade engineering, expansive soils are subjected to long-term wetting–drying cycles, freeze–thaw cycles, and temperature variations. Because repeated wetting–drying cycles, freeze–thaw cycles, and long-term environmental stresses were not evaluated, the long-term mechanical performance, volumetric stability, and fatigue characteristics of C8P0.3 remain unverified; future work should evaluate C8P0.3 under wetting–drying and freeze–thaw cycles. In terms of microstructural characterization, the broad XRD peak at approximately 29–30° may contain overlapping contributions from C-S-H gel and calcite, which cannot be definitively distinguished by XRD alone, and XRD also failed to clearly identify the possible formation of C-A-S-H or calcium aluminate hydrate under alkaline conditions; complementary techniques such as FTIR, TGA/DTG, SEM-EDS, and 29Si NMR are therefore recommended. In addition, SEM observations are qualitative, and quantitative porosity measurements and Ca/Si/Al EDS elemental mapping were not performed; these techniques should be added in future work to better identify reaction products and fiber–matrix interfaces. The effects of fiber orientation distribution and optimal fiber length on the synergistic reinforcement mechanism also remain unclear and warrant further investigation.

## 5. Conclusions

This study used industrial solid waste carbide slag and polypropylene fiber as composite improvement materials. A systematic study on the engineering performance optimization of expansive soil was conducted. A multi-indicator comprehensive evaluation model coupling the CRITIC–entropy weight combination weighting method with the TOPSIS method was constructed. Based on this model, the optimal mix proportion was determined. The main conclusions are as follows:

(1) This study constructed a multi-indicator evaluation model coupling the CRITIC–entropy weight combination weighting method with the TOPSIS method. The model effectively reconciles the inherent contradiction between the CRITIC method’s bias toward indicator conflict and the entropy weight method’s bias toward data dispersion. It provides a decision-making basis for the multi-objective trade-off between swelling–shrinkage and mechanical properties. It also overcomes the preference bias of single weighting methods in comprehensive evaluation.

(2) Based on this model, the optimal mix proportion was determined as C8P0.3 (carbide slag 8%, polypropylene fiber 0.3%). Its relative closeness reaches 0.93, ranking first among the 25 mix proportions. Compared with the untreated soil, this mix proportion reduces the USR by 77.07%. Cohesion and UCS increase by 164.30% and 71.91%, respectively. This achieves synergistic optimization of swelling–shrinkage inhibition and mechanical enhancement. Compared with the single CRITIC–TOPSIS, single entropy weight–TOPSIS, and conventional range analysis methods, the result obtained by this model achieves a better balance between strength and deformation indicators. It avoids the engineering risk of excessive bias toward a single method of measuring performance.

(3) The microscopic mechanism reveals the following. The cementitious products generated by carbide slag hydration fill pores and coat particles. They form a rigid skeleton to effectively restrain swelling–shrinkage deformation. Polypropylene fibers interweave into a three-dimensional network structure within the soil. They provide flexible confinement through interfacial friction. This transforms the failure mode from brittle to ductile. This rigid–flexible dual synergistic effect microscopically explains the fundamental reason. The C8P0.3 mix proportion macroscopically achieves the lowest swelling–shrinkage ratio and the highest comprehensive mechanical score simultaneously.

(4) Several limitations remain. The optimum was determined from 7-day laboratory-cured specimens; long-term durability under wetting–drying and freeze–thaw cycles, leaching behavior, and life-cycle environmental impacts were not evaluated, and microstructural characterization was mainly qualitative. Future work should therefore assess these durability and environmental aspects and apply complementary techniques such as FTIR, TGA/DTG, SEM-EDS, and ^29Si NMR to verify reaction products and fiber–matrix interfaces.

## Figures and Tables

**Table 1:** Physical property indicators of the expansive soil.

Liquid Limit/%	Plastic Limit/%	Plasticity Index/%	Free Swelling Ratio/%	Specific Gravity
59.3	20.9	38.4	68	2.68

**Table 2:** Main chemical composition of carbide slag.

Chemical Composition	SiO_2_	CaO	MgO	Al_2_O_3_	Fe_2_O_3_	SO_3_
Content/%	7.31	84.25	0.17	3.82	0.69	0.74

**Table 3:** Basic physical property indicators of polypropylene fiber.

Type	Length/mm	Diameter/μm	Tensile Strength/MPa	Elastic Modulus/MPa	Acid and Alkali Resistance
Bundled monofilament	12	18~45	≥350	≥3500	Strong

**Table 6:** Pearson correlation matrix of the six evaluation indicators.

Indicator	USR	LSR	VSR	Cohesion	φ	UCS
USR	1.000	0.908	0.975	0.918	0.945	0.916
LSR	0.908	1.000	0.957	0.972	0.939	0.863
VSR	0.975	0.957	1.000	0.973	0.986	0.952
Cohesion	0.918	0.972	0.973	1.000	0.973	0.923
φ	0.945	0.939	0.986	0.973	1.000	0.967
UCS	0.916	0.863	0.952	0.923	0.967	1.000

Note: All correlation coefficients are significant at *p* < 0.01.

**Table 7:** TOPSIS evaluation results.

MP	$C_{i}$	Rank	MP	$C_{i}$	Rank
C0P0	0.00	25	C6P0.3	0.92	3
C0P0.1	0.08	24	C6P0.4	0.88	9
C0P0.2	0.15	23	C8P0	0.80	13
C0P0.3	0.19	22	C8P0.1	0.85	10
C0P0.4	0.25	21	C8P0.2	0.90	6
C4P0	0.50	20	C8P0.3	0.93	1
C4P0.1	0.57	19	C8P0.4	0.90	5
C4P0.2	0.65	18	C10P0	0.79	14
C4P0.3	0.70	16	C10P0.1	0.83	12
C4P0.4	0.70	17	C10P0.2	0.90	4
C6P0	0.76	15	C10P0.3	0.92	2
C6P0.1	0.83	11	C10P0.4	0.88	8
C6P0.2	0.88	7	/	/	/

**Table 8:** Comparison of optimal mix proportions and UCS from different evaluation methods.

Evaluation Method	OMP	USR	LSR	VSR	Cohesion	φ	UCS
Single CRITIC–TOPSIS	C10P0.3	3.54	1.74	0.84	353.88	42.17	2.77
Single Entropy Weight–TOPSIS	C6P0.3	7.95	1.86	0.97	386.25	42.97	2.80
Conventional Range Analysis	C8P0.4	7.81	2.26	1.35	379.57	42.65	2.90
CRITIC–Entropy Weight–TOPSIS	C8P0.3	7.50	1.90	1.19	375.78	43.25	2.82

Note: In the table, “OMP” denotes optimal mix proportion.

## Data Availability

The original contributions presented in this study are included in the article. Further inquiries can be directed to the corresponding author.

## Abbreviations

The following abbreviations are used in this manuscript:

USR	Unloaded Swelling Ratio
LSR	Linear Shrinkage Ratio
VSR	Volumetric Shrinkage Ratio
IFA	Internal Friction Angle
UCS	Unconfined Compressive Strength
C+number	Carbide Slag Content
P+number	Polypropylene Fiber Content
MP	Mix Proportion
OMP	Optimal Mix Proportion

## References

[1] Bai Y., Chang S., Xiao H., Li L., He J., Qiu J., Zhou W., Deng Y. (2024). Research progress in ecological treatment of expansive soil. Chin. J. Geotech. Eng..

[2] Yang Z., Guo R., Ling X., Cai G. (2026). Improvement and engineering application of expansive soils: A review. J. Rock Mech. Geotech. Eng..

[3] Jia W. (2024). The Impact of Cement on the Mechanical Properties of Expansive Soil. J. Civ. Transp. Eng..

[4] Al-Rawas A.A., Hago A.W., Al-Sarmi H. (2005). Effect of lime, cement and Sarooj (artificial pozzolan) on the swelling potential of an expansive soil from Oman. Build. Environ..

[5] Ma B., Cai K., Zeng X., Li Z., Hu Z., Chen Q., He C., Chen B., Huang X. (2021). Experimental Study on Physical-Mechanical Properties of Expansive Soil Improved by Multiple Admixtures. Adv. Civ. Eng..

[6] Shah R., Bhoi M.K., Sharma A.K. (2025). Experimental evaluation of expansive soil stabilised with sustainable binder incorporating blast furnace slag and bagasse ash. Geomech. Geoengin..

[7] Qiu X., Lai Z., Zuo J., Li W. (2025). Synergistic stabilization of expansive soil using ultrafine high-reactivity fly ash and calcium carbide slag: Performance optimization and microstructural insights. Front. Mater..

[8] Dang L.C., Khabbaz H., Ni B.-J. (2021). Improving engineering characteristics of expansive soils using industry waste as a sustainable application for reuse of bagasse ash. Transp. Geotech..

[9] Yang L., Deng X. (2019). Experimental Study on Shear Behavior of Expansive Soil Modified by Gravel Sand. IOP Conf. Ser. Mater. Sci. Eng..

[10] Jun Y., Li X., Zhang G., Tang Y., Yong L. (2013). Expansive Properties of Expansive Soil Improved by Mixing Weathered Sand. J. Yangtze River Sci. Res. Inst..

[11] Liu Y. (2025). Stability analysis of an expansive soil slope under heavy rainfall conditions with different anchor reinforcements. Sci. Rep..

[12] Cheng Y., Xu Y., Wang L., Wang L. (2022). Stability of expansive soil slopes reinforced with anchor cables based on rotational-translational mechanisms. Comput. Geotech..

[13] Huang Y., Zhang H., Shen R., Tan X., Wang X., Wu Y. (2025). Influence of UHMWPE on the expansion characteristics of expansive soil: Experimental studies. PLoS ONE.

[14] Zhang D., Wang Y., Zhang Z., Sun Z., Wang C., Zou S. (2025). Synergistic Control of Shrinkage and Mechanical Properties in Expansive Soil Slurry via Coupled Cement–Fiber Reinforcement. Buildings.

[15] Paul S., Tasnim A., Majumder J. (2025). Enhancement of micro-mechanical characteristics of expansive soil through synergistic incorporation of jute and nylon fibers with cement. Results Eng..

[16] Balcha A., Hassen M., Geremew A., Teshome G. (2025). Engineering properties of expansive soil stabilized with barley husk ash and lime: Case study of Jimma town subgrade soils. Sci. Rep..

[17] Bazarbekova A., Kim Y.R., Little D., Jung J.S., Park Y.B. (2026). Laboratory and field assessment of gypsum-modified aluminosilicate blends for expansive clay stabilization. Transp. Geotech..

[18] Moallemi M., Dehnad M.H., Khodaparast M., Wang X. (2025). Statistical Analysis of Expansive Soil Stabilized With Calcium Carbide Residue and Rice Husk Ash Using Response Surface Methodology. Adv. Civ. Eng..

[19] Blayi R.A., Kakrasul J.I., Hamad S.M. (2025). Optimization and performance of rice husk ash for enhancing unconfined compressive strength at various soil liquid limits using response surface methodology. Prog. Eng. Sci..

[20] Huang K., Xu G., Yan C., Zhang X., Li D., Cai G., Sun Y., Chen R., Pu S., Shi X. (2025). Study on the optimization and performance of expansive soil stabilized by alkali-activated fly ash-phosphogypsum using response surface methodology. J. Build. Eng..

[21] Venkatesh N., Navyasri B., Prasad C.R.V. (2024). Assessing the synergistic effects of GGBS and glass fiber on expansive soil behavior using response surface methodology. J. Build. Pathol. Rehabil..

[22] Zha F., Hu C., Kang B., Qin L., Li J., Chu C. (2024). Formulation of PG-FA-L composite modifier for repairing expansive soil based on the statistical mixed design method. Chemosphere.

[23] Abootalebi S., Hadi-Vencheh A., Jamshidi A. (2019). Ranking the Alternatives With a Modified TOPSIS Method in Multiple Attribute Decision Making Problems. IEEE Trans. Eng. Manag..

[24] Diakoulaki D., Mavrotas G., Papayannakis L. (1995). Determining objective weights in multiple criteria problems: The critic method. Comput. Oper. Res..

[25] Yongfen R., Chunqin G., Zhiwei L., Kewen L. (2017). Cloud model evaluation of swell-shrink grade of swelling soil based on improved analytic hierarchy process and entropy weight method. J. Jiangsu Univ. Nat. Sci. Ed..

[26] Cheng J., Zhou J., Liu J., Zhou Z., Cao X., Cen K. (2003). Microstructure Change in Dynamic Calcination and Sintering of Carbide Slag. J. Chem. Ind. Eng..

[27] Yang Y., Zhu G., Meng Z., Liu X., Yang J., Yan K., Peng Z., Wang Q., Li H. (2023). Research on impurity occurrence and high-efficient separation of dry process calcium carbide slag. Chin. J. Process Eng..

[28] Yaosheng C., Dushan X., Jie X., Feiyue G., Jing Y. (2016). Analysis and Characterization of Physiochemical Property of Carbide Slag andIts Application Study. Shandong Chem. Ind..

[29] Ren G., Fan H., Gao Y., Guo H., Wu T., Zhao G., Zhu Z., Li X., Chun P.-J. (2023). Recycling and conservation of calcium carbide slag in dispersive soil modification: An evaluation of early age performance. Constr. Build. Mater..

[30] (2022). Technical Specification for Buildings in Expansive Soil Regions.

[31] (2020). Test Methods of Soils for Highway Engineering.

[32] (2024). Test Methods of Materials Stabilized with Inorganic Binders for Highway Engineering.

[33] (2015). Technical Guidelines for Construction of Highway Roadbases.

[34] Lijun Z., Neng-wen Y. (2010). Comparison and Selection of Index Standardization Method Linear Comprehensive Evaluation Model. Stat. Inf. Forum.

[35] Lin L., Yunfei W., Zhe H., Xin J. (2023). The Impact of Forestry Ecological Construction Attention on the Environmental Kuznets Curve. For. Eng..

[36] Wen Q.B. (2023). Multi-Index Decision-Making of Service Performance Assessment and Maintenance and Treatment Scheme of Subgrade Slope.

[37] Zeng Z., Lu H., Zhao Y., Qin Y. (2020). Analysis of the mineral compositions of swell-shrink clays from guangxi province, china. Clays Clay Miner..

[38] Gu J., Yang J., Qian H., Wang Y. (2018). Influence of cation onexpansive soil swelling in Nanning. J. Eng..

[39] Chang J., Ma J., Tang X. (2022). Study on Strength Attenuation Characteristics of Residual Expansive Soil under Wetting-Drying Cycles and Low Stress and Its Relationship with Shallow Landslide. Geofluids.

[40] Bouzar B., Pajany Y.M., Abriak Ne Benzerzour M. (2025). Valorization of dredged sediments: Influence of sediment content on mechanical properties and environmental behavior. Green Technol. Sustain..

[41] Bouzar B., Benzerzour M., Abriak N.E. (2024). Evaluation of mineral eco-binders from secondary raw materials: Environmental assessments. J. Mater. Cycles Waste Manag..

